# A suggested approach for imputation of missing dietary data for young children in daycare

**DOI:** 10.3402/fnr.v59.28626

**Published:** 2015-12-17

**Authors:** June Stevens, Fang-Shu Ou, Kimberly P. Truesdale, Donglin Zeng, Amber E. Vaughn, Charlotte Pratt, Dianne S. Ward

**Affiliations:** 1 Department of Nutrition, University of North Carolina, Chapel Hill, NC, USA; 2 Department of Epidemiology, University of North Carolina, Chapel Hill, NC, USA; 3 Department of Biostatistics, University of North Carolina, Chapel Hill, NC, USA; 4 Center for Health Promotion and Disease Prevention, University of North Carolina, Chapel Hill, NC, USA; 5 Clinical Application and Prevention Branch, National Heart, Lung, and Blood Institute, National Institutes of Health, Bethesda, MD, USA

**Keywords:** nutrition, daycare, children, missing data, methods

## Abstract

**Background:**

Parent-reported 24-h diet recalls are an accepted method of estimating intake in young children. However, many children eat while at childcare making accurate proxy reports by parents difficult.

**Objective:**

The goal of this study was to demonstrate a method to impute missing weekday lunch and daytime snack nutrient data for daycare children and to explore the concurrent predictive and criterion validity of the method.

**Design:**

Data were from children aged 2-5 years in the My Parenting SOS project (*n*=308; 870 24-h diet recalls). Mixed models were used to simultaneously predict breakfast, dinner, and evening snacks (B+D+ES); lunch; and daytime snacks for all children after adjusting for age, sex, and body mass index (BMI). From these models, we imputed the missing weekday daycare lunches by interpolation using the mean lunch to B+D+ES [L/(B+D+ES)] ratio among non-daycare children on weekdays and the L/(B+D+ES) ratio for all children on weekends. Daytime snack data were used to impute snacks.

**Results:**

The reported mean (± standard deviation) weekday intake was lower for daycare children [725 (±324) kcal] compared to non-daycare children [1,048 (±463) kcal]. Weekend intake for all children was 1,173 (±427) kcal. After imputation, weekday caloric intake for daycare children was 1,230 (±409) kcal. Daily intakes that included imputed data were associated with age and sex but not with BMI.

**Conclusion:**

This work indicates that imputation is a promising method for improving the precision of daily nutrient data from young children.

The diets of young children are challenging to study because they cannot be expected to supply accurate and precise self-reported information about foods consumed. Multiple 24-h dietary recalls using parents as proxy reporters have been recommended as a feasible method of estimating intake in young children. The standard protocol collects two weekday and one weekend day recall (1). However, parent reports of children's intakes are limited by the fact that many preschool children consume meals and snacks when not in the presence of their parents. In the United States, over 70% of 3- to 5-year-old children are enrolled in some form of non-parental care, and 58% are enrolled in full-day programs (2). Baranowski et al. (3) have shown that mothers with children enrolled in childcare for more than 4.5 h per day are significantly more likely to be unable to report their child's intake during part of the day compared to at-home mothers.

Researchers have used different ways to deal with missing lunch data for children including using only-weekend data (4), limiting study participants to those who reported full days (e.g. non-daycare children), excluding part of the weekday (8 a.m.–5 p.m.) (5), or ignoring and analyzing as complete data (6). An alternative to these methods is imputation, a commonly used strategy for replacing missing data with plausible values that can increase accuracy and decrease bias often caused by missing data. Although imputation of missing data collected via 3-day food records (7) and food frequency questionnaires (8–10) has been reported, we know of no studies that have used imputation to estimate the missing diet data resulting from children's attendance at childcare. The objective of this study is to suggest a method for imputation of missing weekday lunch and daytime snack nutrient data among daycare children.

## Methods

We used baseline data from the My Parenting SOS study (*n=*324), a randomized controlled trial designed to test an intervention promoting parenting practices hypothesized to improve healthy eating and activity behaviors in preschool children. The details of the study design and measurement protocols have been described (11) and are reviewed only briefly here. This convenience sample was recruited in three waves from counties located in central North Carolina. Childcare centers from these areas, particularly those that served low income families, helped distribute recruitment information to families. Eligibility criteria required families to have at least one child between the ages of 2 and 5 years and at least one parent with a body mass index (BMI) greater than 25 kg/m^2^ (based on self-reported height and weight). All study procedures were reviewed and approved by the Institutional Review Board at the University of North Carolina at Chapel Hill.

At in-person measurement visits, a trained and certified data collector measured children's standing height and weight without shoes and in light clothing and recorded child's sex. Parents completed a demographic survey that captured child's age (date of birth) and childcare participation using the following two questions: ‘On average, how many days per week does your 2–5 year old child spend in childcare (care outside the home)?’ and ‘On average, how many hours per day does your 2–5 year old child spend in childcare (care outside the home)?’.

In the 3–4 weeks following this visit, parents completed three days (2 weekdays and 1 weekend day) of unannounced 24-h dietary recalls of the child's intake. Recalls were conducted by certified staff using the Nutrition Data System for Research (NDSR, versions 2009–2010, University of Minnesota, Minneapolis) using traditional multi-pass procedures (12–15). However, parents were not prompted to report foods that their child consumed while in childcare.

The current analysis used the NDSR ‘meal file output’. We extracted the variables for energy (kcal), total carbohydrate (g), total protein (g), total dietary fiber (g), total fat (g), and total sugars (g); hereafter referred to as ‘nutrients’. The day of the week variable was collapsed into two categories: ‘weekday’ (Monday to Friday) or ‘weekend’ (Saturday, Sunday). Using the NDSR meal name code, we defined eating occasion as breakfast, lunch, dinner, or snack. Meals that were coded as ‘other’ in NDSR were included in the snack category. Daytime snack was defined as a snack consumed between 8 a.m. and 5 p.m. Evening snack (ES) was defined as a snack consumed anytime outside the 8 a.m. to 5 p.m. window. The eating location variable was categorized as ‘childcare’ or ‘daycare’ if either of the following conditions was met: 1) eating occasion location in NDSR was reported as either childcare or school or 2) the parent reported that the child attended childcare at least five days a week for at least five hours per day and the child had no lunch reported on a weekday. If the conditions were not met, then the eating location was categorized as ‘non-childcare’ or ‘non-daycare’.

The analytic sample and the number of recalls provided by each child are detailed in Supplementary File 1. Data from children missing age (*n*=5), sex (*n*=1), or all three dietary recalls (*n*=10) were excluded. The analysis sample included 308 children with 870 days of dietary recalls. The majority (85.7%) of the children had three dietary recalls. There were 369 weekday recalls in which the child was in daycare, 215 weekday recalls in which the child was not in daycare, and 286 weekend recalls. Not all children provided both weekend and weekday recalls, and some children contributed recalls in daycare and outside of daycare. Weekday lunch data were reported by the parent for four children in daycare (five recalls). The information obtained directly from the parent on their child's intake will be called ‘reported’ to distinguish from data that are imputed.

### Statistical methods

The imputation of missing weekday lunch data for daycare children was based on an interpolation of model-predicted weekend lunch intake for all children and the model-predicted weekday lunch intake for non-daycare children, with respect to their breakfast, dinner, and evening snack (B+D+ES) intake. This approach is valid under the assumption that the missing mechanism is missing at random. Since the missing values are due to some children attending daycare and the sample was relatively homogenous in terms of being low income, we determined that the missing at random assumption is likely to hold.

In the first step, multivariate linear mixed effects models were used to infer the predicted distribution of the missing lunch and daytime snack given all reported data, where the child's age, sex, and BMI were controlled, within-subject dependence was accounted for, and child-specific random effects were included. We did not include the five weekday daycare lunch intakes in these models. Since the nutrient intakes were highly right-skewed, we transformed the data using natural logarithms to obtain more normally distributed data. One multivariate model was fitted for each nutrient with the three outcomes being intake at 1) breakfast, dinner, and evening snacks, 2) lunch, and 3) daytime snack. All models controlled for day of the week (weekday or weekend), eating location (childcare or non-childcare), age, age squared, sex, and BMI. Age and BMI were centered at their means, 42 months and 16 kg/m^2^, respectively. Additional details on the imputation model are in Supplementary File 2.

In the second step, because we did not have information on weekday daycare lunch intake, we used weekend intake and weekday home intake information to infer the weekday daycare lunch intake on the log scale. We explored five weight pairs to evaluate the impact of giving different amounts of influence to weekend intake of all children versus weekday intake of non-daycare children. It was assumed that for daycare children the proportion of their weekday lunch nutrient intake (the unknown) to their breakfast, dinner, and evening snack intake [log(L)/log(B+D+ES)] was equal to the weighted sum of the log(L)/log(B+D+ES) ratio from weekend days for all children plus the log(L)/log(B+D+ES) ratio from weekdays for non-daycare children [i.e. log(L)/log(B+D+ES) ratio from weekday in childcare equal to $k\frac{\alpha_{2}}{\alpha_{1}}+(1-k)\frac{\alpha_{2}+\beta_{2}}{\alpha_{1}+\beta_{1}}$, where *k* is a weight parameter which is between 0 and 1]. The weights (*k*, 1–*k*) were determined by the prior belief of whether the weekday daycare intake was more similar to a weekday home intake or a weekend intake. A *k* value greater than 0.5 indicates a prior belief that the weekday daycare intake is more similar to a weekend intake than a weekday home intake; on the other hand, a *k* value smaller than 0.5 indicates a prior belief that the weekday daycare intake is more similar to a weekday home intake than a weekend intake. For this evaluation, we used five pairs of weights (*k*, 1–*k*) as multipliers prior to calculating the sum: 0 and 1; 0.25 and 0.75; 0.5 and 0.5; 0.75 and 0.25; and 1 and 0. The greater the weight used with a ratio, the greater the impact of that ratio on the summed value. Thus, the difference in intake between ‘weekday in childcare’ and ‘weekday not in childcare’ for an average 42-month-old girl with a BMI of 16 kg/m^2^ for lunch could be calculated.

The third step was to impute the missing weekday daytime snack for daycare children. Weights similar to those in step 2 were not necessary for this imputation because weekday daytime snack information was partially available for daycare children. Therefore, we could estimate the parameter γ_3_ and directly used the parameter estimates from the model described in step 1 to predict weekday daytime snack intake for daycare children.

In the fourth step, after the coefficients were estimated using mixed models and the difference in intake between ‘weekday in childcare’ and ‘weekday not in childcare’ for lunch for an average child was calculated, we generated a child-specific predicted distribution of lunch and daytime snack for each child conditional on their individual B+D+ES intake on a specific intake day and child-specific random effect. We randomly drew five sets of final imputed lunch and daytime snack from the child-specific posterior distributions of the lunch and daytime snack intake conditional on child's B+D+ES intake. We then transformed the nutrients back to their original scale by taking the exponential.

We conducted preliminary explorations of the validity of our imputation in two ways. First, we compared the reported and imputed weekday childcare lunch nutrient intakes for the five days for which the reported and imputed data were both available. We used this analysis as a demonstration of a method to assess criterion validity. Second, we examined the concurrent predictive validity by comparing the associations of age, sex, and BMI with energy intake with and without inclusion of imputed data. For the model using imputed data, we analyzed the data following standard analysis procedures for multiple imputed dataset. All statistics were performed using SAS software (version 9.3; SAS Institute, Cary, NC).

## Results

Children's mean age was 42 months (~3.5 years) and almost half were girls (Table 1). The mean BMI was 16 kg/m^2^. Over a third of the sample (37.7%) was African-American and a small percentage (5.8%) was Hispanic.

**Table 1:** Demographic characteristics and dietary intakes of the overall study sample and for weekend, weekday non-daycare, and weekday daycare children

	Study sample (*n*=308)	Weekend all children (*n*=284)	Weekday non-daycare children (*n*=121)	Weekday daycare children (*n*=202)
Age [months, mean (SD)]	41.7 (10.3)	41.6 (10.0)	41.3 (11.3)	41.9 (9.6)
Sex (% girls)	48.4	48.2	48.8	45.5
Weight [kg, mean (SD)]	16.1 (3.0)	16.1 (3.0)	15.9 (2.7)	16.3 (3.2)
Height [cm, mean (SD)]	98.8 (7.8)	98.8 (7.7)	98.8 (8.0)	99.0 (7.8)
BMI [kg/m^2^, mean (SD)]	16.3 (1.5)	16.4 (1.5)	16.2 (1.4)	16.5 (1.5)
BMI percentile [mean (SD)]	60.7 (28.2)	60.8 (28.4)	58.8 (29.4)	62.8 (26.8)
Hispanic (%)	5.8	6.0	3.3	7.4
Race (%)				
African-American	37.7	37.3	38.0	38.6
Caucasian	50.7	51.4	50.4	50.5
Others/missing	11.7	11.3	11.6	10.9

Nutrient information is shown for meals and snacks as reported (Table 2). We found that 63.2% of weekday recalls were from daycare children and were missing lunch and daytime snack data. Combined breakfast, dinner, and evening snack energy intakes were similar for all children on the weekend, non-daycare children on weekdays, and daycare children on weekdays (668, 662, and 679 kcal, respectively). Nutrients from lunch and daytime snacks on weekends were similar to those from lunch and daytime snacks on weekdays for non-daycare children. For daycare children, information on weekday lunch and daytime snacks were reported in only 5 and 87 recalls, respectively. The mean (± standard deviation) energy intakes for weekday lunches [313 kcal (±145)] and daytime snacks [176 kcal (±136)] in the limited number of recalls from daycare children were lower than the energy intakes from lunch and daytime snacks on weekend days [276 kcal (±213) and 229 kcal (±219), respectively] and from weekdays in non-daycare children [224 kcal (±226) and 162 kcal (±181), respectively].

**Table 2:** Reported dietary intakes of the overall study sample and for weekend, weekday non-daycare, and weekday daycare children

	Weekend all children	Weekday non-daycare children	Weekday daycare children
Energy [kcal, mean (SD)]			
Total	1,173 (427)	1,048 (463)	725 (324)
Breakfast+dinner+evening snack	668 (316)	662 (317)	679 (304)
Lunch	276 (213)	224 (226)	313^a^ (145)
Daytime snack	229 (219)	162 (181)	176^a^ (136)
Carbohydrate [g, mean (SD)]			
Total	166.1 (65.1)	146.6 (70.1)	103.1 (49.9)
Breakfast+dinner+evening snack	92.4 (45.6)	91.2 (48.9)	95.4 (46.0)
Lunch	35.4 (29.3)	29.1 (31.9)	46.9^a^ (27.4)
Daytime snack	38.3 (35.5)	26.3 (28.7)	30.1^a^ (24.2)
Protein [g, mean (SD)]			
Total	41.8 (17.4)	39.8 (19.6)	27.7 (13.3)
Breakfast+dinner+evening snack	25.6 (13.8)	27.0 (15.0)	26.6 (12.7)
Lunch	10.5 (8.7)	8.7 (9.0)	14.8^a^ (7.4)
Daytime snack	5.7 (7.3)	4.1 (6.0)	4.0^a^ (4.2)
Fiber [g, mean (SD)]			
Total	9.5 (5.2)	9.9 (8.0)	6.1 (4.4)
Breakfast+dinner+evening snack	5.4 (3.7)	6.2 (5.7)	5.6 (3.7)
Lunch	2.2 (2.3)	2.2 (4.2)	3.2^a^ (3.1)
Daytime snack	1.9 (2.6)	1.6 (2.2)	2.0^a^ (4.8)
Fat [g, mean (SD)]			
Total	40.5 (20.2)	36.0 (19.4)	23.8 (14.3)
Breakfast+dinner+evening snack	23.1 (14.7)	22.4 (13.5)	22.5 (13.7)
Lunch	10.7 (10.7)	8.5 (9.7)	7.9 (3.3)
Daytime snack	6.7 (9.5)	5.0 (7.3)	5.1 (5.4)
Sugar [g, mean (SD)]			
Total	86.9 (40.1)	74.1 (43.0)	51.9 (27.1)
Breakfast+dinner+evening snack	47.3 (25.8)	45.0 (28.7)	47.9 (24.7)
Lunch	16.1 (14.7)	14.5 (18.1)	24.9 (17.9)
Daytime snack	23.5 (24.1)	14.7 (17.9)	15.8 (14.5)
Added sugar [g, mean (SD)]			
Total	46.9 (34.9)	37.7 (31.0)	27.3 (22.3)
Breakfast+dinner+evening snack	26.0 (23.7)	22.9 (22.8)	24.7 (20.9)
Lunch	8.3 (11.9)	7.1 (11.5)	15.8 (16.7)
Daytime snack	12.6 (17.9)	7.7 (11.8)	10.0 (12.3)
Calcium [mg, mean (SD)]			
Total	742.7 (392.7)	687.1 (434.6)	480.6 (281.6)
Breakfast+dinner+evening snack	464.4 (275.2)	466.5 (321.8)	456.2 (271.9)
Lunch	145.5 (155.0)	124.2 (150.3)	237.7 (187.0)
Daytime snack	132.7 (186.2)	96.4 (163.6)	89.7 (117.1)

a Five of the 369 recalls from daycare children on weekdays included data on lunch and 87 included data on daytime snacks.

We found that the impact of using different weights as multipliers in the imputation process was small because the weekend log(L)/log(B+D+ES) ratio and the weekday non-daycare ratio were very similar for all nutrients. For example, for total energy the weekend ratio was 0.9041 and the weekday non-daycare ratio was 0.9056. The largest difference between the two ratios, albeit still relatively small, was for fiber (0.5762 for weekend ratio and 0.6110 for weekday non-daycare ratio). We therefore used the same weight for each multiplier (0.5) such that the weekday log(L)/log(B+D+ES) ratio for daycare children was the average of the weekend and the weekday non-daycare log(L)/log(B+D+ES) ratios.

As expected, after imputation the mean daily intakes for all nutrients increased (Table 3). For daycare children, the imputation resulted in adding (on average) 505 kcal to their daily weekday intake (382 kcal from lunch and 123 kcal from snacks). In general, imputation increased the mean intake of carbohydrate by 69.7 g, protein by 17.9 g, fiber by 4.7 g, fat by 19.2 g, and sugar by 38.9 g for weekday daycare recalls. After combining the reported and imputed data, the overall increases in the mean intakes across all days were smaller (carbohydrate 29.6 g, protein 7.6 g, fiber 2.1 g, fat 8.1 g, and sugar 16.6 g).

**Table 3:** Mean (standard deviation) nutrient level in the reported and imputed datasets for weekday daycare recalls, weekday for all children, and overall for all children

Nutrient	Weekday daycare (*n=*369 recalls)			Weekday recalls (*n*=584 recalls)			All available recalls (*n*=870 recalls)		
		
	Reported data	Imputed and reported data		Reported data	Imputed and reported data		Reported data	Imputed and reported data	
		
	Mean (SD)	Mean (SD)	Diff^a^	Mean (SD)	Mean (SD)	Diff	Mean (SD)	Mean (SD)	Diff
Energy (kcal)	725 (324)	1,230 (409)	505	839 (351)	1,161 (384)	322	948 (315)	1,163 (335)	215
Carbohydrate (g)	103.1 (49.9)	172.8 (63.1)	69.7	118.2 (53.1)	162.4 (58.0)	44.2	133.8 (48.3)	163.4 (51.1)	29.6
Protein (g)	27.7 (13.3)	45.6 (17.4)	17.9	32.1 (14.2)	43.5 (15.3)	11.4	35.2 (12.6)	42.8 (13.2)	7.6
Fiber (g)	6.1 (4.4)	10.8 (5.9)	4.7	7.4 (4.9)	10.5 (5.3)	3.1	8.1 (4.4)	10.2 (4.6)	2.1
Fat (g)	23.8 (14.3)	43.0 (23.2)	19.2	28.2 (14.6)	40.4 (17.1)	12.2	32.3 (13.6)	40.4 (15.4)	8.1
Sugar (g)	51.9 (27.1)	90.8 (55.1)	38.9	59.7 (30.4)	84.7 (44.0)	25.0	68.5 (28.6)	85.1 (36.8)	16.6
Added sugar (g)	27.3 (22.3)	61.1 (75.0)	33.8	30.7 (21.9)	52.2 (51.1)	21.5	35.9 (22.8)	50.3 (39.4)	14.4
Calcium (mg)	480.6 (281.6)	756.1 (417.5)	275.5	557.4 (313.2)	736.5 (366.4)	179.1	617.8 (293.4)	734.9 (324.5)	117.1

a Diff (Difference), imputed and reported data minus reported data.

**Fig. 1:**
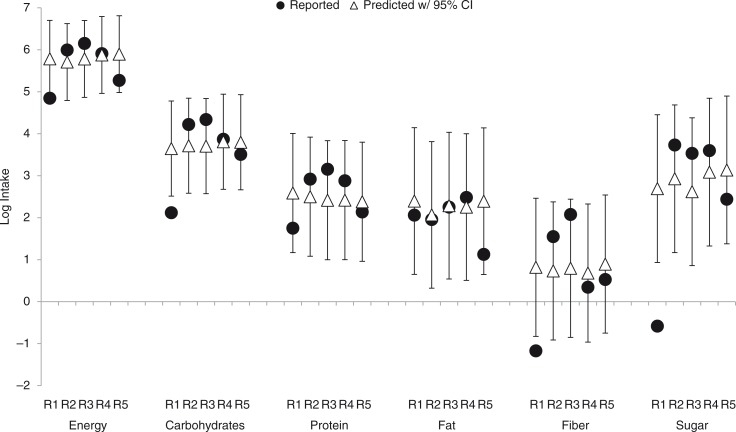
Examination of the criterion validity of the imputation by comparing the predicted mean and 95% confidence interval of lunch intake to reported data in the five weekday daycare children recalls (four children) with reported lunch data (R4 and R5 are from the same child). The solid circles indicate the reported log intake and triangles with vertical bars represent the posterior mean log intake and 95% confidence interval. Weight parameter pair was set to 0.5, 0.5.

The five recalls that included reported weekday lunch from daycare children were used to examine the criterion validity of the imputation by comparing the reported nutrient values to the posterior mean nutrient values and the corresponding 95% CI (Fig. 1). The reported log intake was within the 95% CI of the posterior mean for all recalls for protein and fat. For one recall (R1), the reported data were slightly outside of the 95% CI with the imputation overestimating the energy, carbohydrate, fiber, and sugar intake. The actual reported intake for this recall was 127 kcal, 8.3 g carbohydrates, 0.3 g fiber, and 0.6 g sugar compared to the imputed intake (and 95% CI) of 326 kcal (95% CI: 130, 814), 38.4 g carbohydrates (95% CI: 12.4, 119.3), 2.3 g fiber (95% CI: 0.4, 11.8), and 14.8 g sugar (95% CI: 2.5, 86.0).

To examine concurrent predictive validity, we examined the association of total energy intake with age, sex, and BMI using four different approaches to handling the missing data due to attendance at childcare (Table 4). As expected, when only partial day data were used (removing foods eaten between 8 a.m. and 5 p.m.) the total energy intake was low (633.8 kcal). In comparison, using weekend-only data resulted in a mean energy intake of 1,124.8 kcal for a 42-month-old girl with a BMI of 16 kg/m^2^. If the full-weekend data and only-weekday non-daycare data were used, the mean energy intake was 1,080.5 kcal. After imputation, total energy intake was intermediate between the latter two values at 1,099.1 kcal.

**Table 4:** Comparisons of the associations of age, sex, and BMI with energy intake in four dietary datasets using reported and imputed data in order to assess the concurrent predictive validity of the imputation

	Reported						Imputed and Reported^a^	
	
	Partial weekend and weekday^b^ (*n=*870 recalls in 308 children)		Weekend only (*n*=286 recalls in 284 children)		Weekend and partial weekday^c^ (*n*=501 recalls in 294 children)		Weekend and weekday (*n*=870 recalls in 308 children)	
			
	Estimate	*p*	Estimate	*p*	Estimate	*p*	Estimate	*p*
Intercept	633.8	<0.001	1,124.8	<0.001	1,080.5	<0.001	1,099.1	<0.001
Age (months)^d^	5.2	<0.001	2.8	0.284	2.7	0.236	5.1	0.007
Sex (boy)	66.5	0.009	95.5	0.060	91.2	0.048	113.2	0.003
BMI (kg/m^2^)^d^	11.7	0.174	5.0	0.767	11.6	0.452	12.1	0.333

a All reported weekend data and reported and imputed weekday data.

b All recall data with 8 a.m. to 5 p.m. intake subtracted from all recalls (weekdays and weekend days).

c All weekend data and partial weekday day (excluded data from daycare children).

d Model coefficients with age centered at 42 months and BMI centered at 16 kg/m^2^.

Age was associated with energy intake in the dataset that excluded all data collected between 8 a.m. and 5 p.m. and in the dataset with missing data imputed. The associations of sex with energy intake were larger and had smaller *p*-values when the imputed data were included. Coefficients indicated energy intake increased 5.1 kcal per month of age and that boys consumed an average of 113.2 kcal more than girls. The association with BMI was not significant in any of the datasets after controlling for age and sex.

## Discussion

Although imputation is a commonly used method for handling missing data, to our knowledge it had not previously been applied to address missing data in children's diet data caused by food consumed while away from the parent (e.g. attending childcare). The few studies that have used imputation for missing diet data have generally estimated intakes of select foods (e.g. fruit, sweets and snacks, milk, tomato products) from incomplete food frequency surveys (16–18) or food records (7). In the current study, 63.2% of weekday recalls were from daycare children and were missing lunch and daytime snack data. For these children, imputation of missing data increased their mean usual intake by 505 kcal, 69.7 g carbohydrates, 17.9 g protein, 4.7 g fiber, 19.2 g fat, 38.9 g sugar, 33.8 g added sugar, and 275.5 mg calcium. Imputed results provided intake estimates more similar to those for children who had full-day diet data. Furthermore, assessment of concurrent predictive validity demonstrated expected associations of energy intake with child age and sex. The lack of association between energy intake and child BMI observed is not uncommon, particularly in studies with young children and self-reported diet data (19–25). Previous studies have found associations between sedentary activities (2, 20) and moderate to vigorous physical activities (22) and BMI in children. It has been shown that the majority of mothers of 3- to 5-year-olds who were not at home during the day were unable to provide full-day information about their child's intake (37% provided no information and 15% only partial information) (3). Direct observation of foods eaten at childcare has been conducted by researchers to reduce missing data (26, 27). Such methods are expensive and often not feasible. An alternate approach adopted by several national surveillance surveys (28–30) is to flag missing meal data and conduct follow-up interviews with childcare providers. However, Briefel et al. (31) showed that enhancing parent-reported recalls with other caregiver reports produced results similar to those of unenhanced protocols (1,159 kcal/day±28.5 vs. 1,131 kcal/day±33.5). Other researchers have addressed the missing data issue by eliminating data from any days in which the parent was unable to report one or more of their child's main meals (i.e. breakfast, lunch, dinner) (5, 32, 33). Studies using this approach have generally eliminated 10–27% of the sample; however, this approach would have excluded 63% of our weekday recalls.

Our imputation study is based on several assumptions. Especially important was the assumption that the models described in step 1 can accurately predict the unmeasured lunch data for childcare children. These models depend on the assumption that the parent-reported food intake data were complete and accurate and that the weekday lunch intakes of daycare children are related to intakes of non-daycare children on weekdays and all children on weekends. We also assumed that the weekday daycare children's log(L)/log(B+D+ES) ratio was at the mean of the log(L)/log(B+D+ES) ratios for all children's weekend day intakes and non-daycare children's weekday intakes (observed to be very similar in our study). This last assumption should be confirmed before applying this method to other samples. For example, if children bring packed lunch to school then the parent would know what food was provided but might not know how much was consumed. In comparison, if children ate lunches provided at school then the parent is dependent on the lunch menu to know what food was provided as they might not know how much their child consumed otherwise. Finally, the missing at random assumption will depend upon study context, and its applicability should be judged accordingly.

One strength of this study is that the majority of the children have three recalls (two weekdays and one weekend day). This is important for estimating average daily intake given that weekday and weekend day intakes are known to be different in older children and adults (34–36). Also, the imputation used multivariate linear mixed effects models which took into account the within-subject and between eating occasion dependence. Because of the small sample size (*n*=5), we must view our examination of the criterion validity of the imputation as a demonstration of the method and not conclusive. Future work that includes highly valid measures of foods eaten at childcare in an adequately sized sample of children can follow the methods outlined here to provide criterion validity of the imputation results.

Observed or reported data are almost always strongly preferred over imputed data; however, young children and their parents who are not present at the child's meal cannot be expected to provide accurate reports of foods consumed. This study offers imputation as an alternate strategy of handling missing or inaccurate data from parent reports of child intake during childcare. Although more work is needed to validate this approach, imputation is likely preferable to methods currently used when proxy observation and reports of dietary intakes of children while in childcare is not feasible. It is our hope that this demonstration of an imputation method applied specifically to this problem will support future work by other investigators to move this field forward.

## Supplementary Material

A suggested approach for imputation of missing dietary data for young children in daycare

A suggested approach for imputation of missing dietary data for young children in daycare
