# Supplementary material for: A suggested approach for imputation of missing dietary data for young children in daycare
Source: Food Nutr Res. 2015 Dec 17;59:10.3402/fnr.v59.28626. doi: 10.3402/fnr.v59.28626 (PMC4685296; doi:10.3402/fnr.v59.28626)
Supplement: A suggested approach for imputation of missing dietary data for young children in daycare [file FNR-59-28626-s001.docx]

Supplemental Material 1

Exclusion criteria and description of amount and type of dietary data.

324 Children

308 Children

Exclusions:
Missing age (n=5)
Missing sex (n=1)
Missing all 3 dietary recalls (n=10)

1 dietary recalls:
10 Children

2 dietary recalls:
34 Children

3 dietary recalls:
264 Children

2 weekend,
1 weekday child care
recalls: 1 child (10)

1 weekend,
2 weekday child care
recalls: 155 children (11)

1 weekend,
1 weekday child care
1 weekday non-child care
recalls: 17 children (12)

1 weekend,
2 weekday non-child care
recalls: 89 children (13)

3 weekday child care
recalls: 1 child (14)

2 weekday child care
1 weekday non-child care
recalls: 1 child (15)

2 weekend
recalls: 1 child (4)

1 weekend,
1 weekday child care
recalls: 12 children (5)

1 weekend,
1 weekday non-child care
recalls: 5 children (6)

2 weekday child care
recalls: 9 children (7)

1 weekday child care
1 weekday non-child care
recalls: 2 children (8)

2 weekday non-child care
recalls: 5 children (9)

1 weekend
recalls: 4 children (1)

1 weekday child care
recalls: 4 children (2)

1 weekday non-child care
recalls: 2 children (3)
