# Supplementary material for: A suggested approach for imputation of missing dietary data for young children in daycare
Source: Food Nutr Res. 2015 Dec 17;59:10.3402/fnr.v59.28626. doi: 10.3402/fnr.v59.28626 (PMC4685296; doi:10.3402/fnr.v59.28626)
Supplement: A suggested approach for imputation of missing dietary data for young children in daycare [file FNR-59-28626-s002.doc]

Supplemental Material 2

Modeling details

One multivariate model was fitted for each nutrient with the three outcomes being intake at 1) breakfast, dinner and evening snacks (B+D+ES), 2) lunch and 3) daytime snack. All models controlled for day of the week (weekday or weekend), eating location (child care or non-child care), age, age squared, gender and BMI. Age and BMI were centered at their means, 42 months and 16 kg/m^2^, respectively. Specifically, the model was as follows,

where $i$ indexes the child and $j$indexes the recall for each child. $y_{1ij}$, $y_{2ij}$, and $y_{3ij}$ are the breakfast, dinner and evening snacks (B+D+ES) intake, the lunch intake, and the daytime snack intake for child $i$ at the $j^{th}$ recall, respectively. $z_{1ij}$, $z_{2ij}$, and $z_{3ij}$ are the natural logarithm of $y_{1ij}$, $y_{2ij}$, and $y_{3ij}$, respectively. $W_{ij}$is the weekday indicator which takes on value 1 if the $j^{th}$ intake of child $i$ was on a weekday and 0 otherwise. $D_{ij}$is the child care indicator which takes on value 1 if the $j^{th}$ intake of child $i$ was in child care and 0 otherwise. $\varepsilon_{1ij}$, $\varepsilon_{2ij}$, and $\varepsilon_{3ij}$ are the multivariate error terms specific to a child on a given day which is jointly normally distributed

($\left(\begin{matrix} \varepsilon_{1ij} \\ \varepsilon_{2ij} \\ \varepsilon_{3ij} \end{matrix}\right)\sim N(\left(\begin{matrix} 0 \\ 0 \\ 0 \end{matrix}\right),\left(\begin{matrix} \sigma_{11} & \sigma_{21} & \sigma_{31} \\ \sigma_{21} & \sigma_{22} & \sigma_{32} \\ \sigma_{31} & \sigma_{32} & \sigma_{33} \end{matrix}\right))$ ). $a_{i}$are child specific random effects which are normally distributed ($a_{i}\sim N(0,\sigma_{a}^{2}$)) and independent of $\varepsilon_{1ij}$, $\varepsilon_{2ij}$, and $\varepsilon_{3ij}$. Coefficients $\alpha_{1}$, $\alpha_{2}$, and $\alpha_{3}$ represent natural logarithm of B+D+ES intake, lunch intake, and daytime snack intake for an average 42-month old girl with 16 BMI during weekend, respectively. Coefficients $\beta_{1}$, $\beta_{2}$, and $\beta_{3}$ represent the differences in natural logarithm of intake between “weekday not in child care” and “weekend” for an average 42-month old girl with 16 BMI on B+D+ES, lunch, and daytime snack, respectively. Coefficients $\gamma_{1}$and $\gamma_{3}$ represent the differences in natural logarithm of intake between “weekday in child care” and “weekday not in child care” for an average 42-month old girl with 16 BMI on B+D+ES and daytime snack, respectively. Coefficients $\theta_{1,age}$, $\theta_{2,age}$, $\theta_{3,age}$, $\theta_{1,age^{2}}$, $\theta_{2,age^{2}}$, and $\theta_{3,age^{2}}$represent the linear and quadratic age effects for an average girl with 16 BMI on natural logarithm intake of B+D+ES, lunch, and daytime snack, respectively. Coefficients$\theta_{1,boy}$, $\theta_{2,boy}$, and $\theta_{3,boy}$ represent the differences in natural logarithm intake for boys vs. girls on B+D+ES, lunch, and daytime snack, respectively. Coefficients$\theta_{1,BMI}$, $\theta_{2,BMI}$, and $\theta_{3,BMI}$ represent the effects of one unit increase in BMI for an average 42-month old girl on natural logarithm intake of B+D+ES, lunch, and daytime snack, respectively.
